# Long Non-Coding RNAs in Epithelial-Mesenchymal Transition of Pancreatic Cancer

**DOI:** 10.3389/fmolb.2021.717890

**Published:** 2021-11-08

**Authors:** Kenji Takahashi, Kenzui Taniue, Yusuke Ono, Mikihiro Fujiya, Yusuke Mizukami, Toshikatsu Okumura

**Affiliations:** ^1^ Division of Metabolism and Biosystemic Science, Gastroenterology and Hematology/Oncology, Department of Medicine, Asahikawa Medical University, Asahikawa, Japan; ^2^ Isotope Science Center, The University of Tokyo, Bunkyo, Japan; ^3^ Institute of Biomedical Research, Sapporo Higashi Tokushukai Hospital, Sapporo, Japan

**Keywords:** long non-coding RNA (lncRNA), extracellular vesicles (EVs), pancreatic ductal adenocarcinoma (PDAC), epithelial-mesenchymal transition (EMT), liquid biopsy, microRNA (miRNA)

## Abstract

Non-coding RNAs (ncRNAs), or RNA molecules that do not code for proteins, are generally categorized as either small or long ncRNA (lncRNA) and are involved in the pathogenesis of several diseases including many cancers. Identification of a large number of ncRNAs could help to elucidate previously unknown mechanisms in phenotype regulation. Some ncRNAs are encapsulated by extracellular vesicles (EVs) and transferred to recipient cells to regulate cellular processes, including epigenetic and post-transcriptional regulations. Recent studies have uncovered novel molecular mechanisms and functions of lncRNAs in pancreatic ductal adenocarcinoma (PDAC), one of the most intractable cancers that is highly invasive and metastatic. As the epithelial-mesenchymal transition (EMT) triggers tumor cell invasion and migration, clarification of the roles of lncRNA in EMT and tumor cell stemness would be critical for improving diagnostic and therapeutic approaches in metastatic cancers. This review provides an overview of relevant studies on lncRNA and its involvement with EMT in PDAC. Emerging knowledge offers evidence for the dysregulated expression of lncRNAs and essential insights into the potential contribution of both lncRNAs and EVs in the pathogenesis of PDAC. Future directions and new clinical applications for PDAC are also discussed.

## Introduction

Pancreatic ductal adenocarcinoma (PDAC) is the most common type of pancreatic cancer. It is one of the most dismal cancers as the associated 5-years relative survival rate of PDAC is only approximately 10%. The fundamental issue related to the significant mortality is the difficulty of diagnosis at the early stage (Ying et al., 2016; Lai et al., 2019), which leads to more than half of cases being diagnosed at advanced stages. Additionally, there is a trend toward an increase in PDAC incidence every successive year (Siegel et al., 2017). Surgical resection is the only curative treatment for PDAC to date. However, less than 20% of cases are potentially curable due to the persistent invasion and distant metastasis (Hidalgo 2010). Chemotherapeutic agents, such as gemcitabine, fluorouracil, irinotecan, oxaliplatin, and various nanoparticles, have been used widely to treat unresectable PDAC; however, the efficacy of these treatments is modest owing to frequent metastasis (Kamisawa et al., 2016; Brunetti et al., 2018). Behaviors causing the high malignant potential of PDAC, such as tumor cell invasion, metastasis, and chemoresistance, are associated with epithelial-mesenchymal transition (EMT) (De Craene and Berx 2013; Zhou et al., 2017). During this process, drastic alterations in gene expression can be induced that disrupt epithelial properties and lead to a mesenchymal phenotype. Various factors can induce EMT, including transforming growth factor (TGF)-β, which can induce EMT-associated transcription factors, such as the zinc finger transcription factor Snail, subsequently conferring tumor cell migration, metastasis, and resistance to cell death and chemotherapy in several types of cancers (De Craene and Berx 2013; Turley et al., 2008; Zhou et al., 2017). Thus, an urgent need exists to investigate regulatory mechanisms of EMT and identify molecular targets to suppress invasion, metastasis, and resistance to chemotherapy in PDAC patients.

To date, massive parallel sequencing studies have identified several non-protein coding RNAs (ncRNAs). NcRNAs play important roles in gene or protein regulation and cellular activity (Anastasiadou et al., 2018; Slack and Chinnaiyan 2019). Among them, long non-coding RNAs (lncRNAs) are increasingly recognized to govern substantial biological processes in multiple cancers through diverse mechanisms, such as epigenetic regulation (Rinn and Chang 2012). While well-known lncRNAs, such as *H19*, Metastasis associated lung adenocarcinoma transcript 1 (*MALAT1*), or HOX antisense intergenic RNA (*HOTAIR*), are reported as pan-cancer markers across different tissues, newly discovered lncRNAs [e.g., long intergenic non-coding RNA 01111 (*LINC01111*) in PDAC] have also been shown to work as biomarkers for several cancers (Pandya et al., 2020; Takahashi, Yan et al., 2014a). Some of the lncRNAs, such as *MALAT1*, highly upregulated in liver cancer (*HULC*) or urothelial carcinoma-associated 1 (*UCA1*), can be encapsulated in extracellular vesicles (EVs) and, when transferred, affect cell signaling and phenotypes similar to microRNAs (miRNAs) (Kogure et al., 2011; Takahashi et al., 2014c; Tkach and Thery 2016; Valadi et al., 2007). Although the underlying molecular mechanisms by which lncRNAs control PDAC initiation and progression are not well understood, a subset of the lncRNAs, transferred by EVs, is reported to be involved in the regulation of EMT to mediate tumor invasion and metastasis (Duguang et al., 2017; Xu et al., 2020). There are several factors, such as the investigation of EMT regulation by lncRNAs, the validation of diagnostic methods using candidate lncRNAs, and quantitation and standardization of technical issues for the diagnostic methods, that should be described before attempting the use of lncRNAs in clinical applications (Pandya et al., 2020). Therapeutic interventions for pancreatic cancer using lncRNA-based strategies can be designed based on the existing and emerging knowledge on lncRNAs in PDAC (Crowley et al., 2013; Liang et al., 2017; Shukla et al., 2018).

Here, we describe the features of lncRNAs produced from intergenic loci and the current knowledge regarding EMT in PDAC. We also present the roles of oncogenic and tumor-suppressive lncRNAs in EMT regulation in PDAC, as well as possible strategies to utilize the lncRNAs associated with EMT to develop clinical applications for diagnosis and therapy of human PDAC.

## Long Non-Coding RNA

The Functional ANnoTation Of the Mammalian Genome (FANTOM) consortium discovered and reported on numerous mammalian transcriptomes which do not code for proteins and are defined as non-protein coding RNAs by sequencing novel full-length mouse cDNAs (Okazaki et al., 2002; Carninci et al., 2005). A similar but different project, the Encyclopedia of DNA Elements (ENCODE) project, advanced sequence-based studies to map functional elements across the human genome, revealing that RNAs transcribed from more than 80% of the genome, including ncRNAs, would have biochemical functions (Derrien et al., 2012; Dunham et al., 2012). NcRNAs are generally divided into two groups based on their base length, small ncRNA (<200 bp) and lncRNA (>200 bp). Small ncRNAs include miRNA, transfer RNA-derived small RNAs, or PIWI-interacting RNAs (Slack and Chinnaiyan 2019). Meanwhile, lncRNAs play critical roles in diverse biological processes, including initiation of cellular differentiation, proliferation, and pluripotency (Hu et al., 2012; Kopp and Mendell 2018; Takahashi et al., 2014a; Taniue and Akimitsu 2021). The subcellular localization of lncRNAs is as diverse as that observed with protein coding mRNAs. While mRNAs are mainly localized to the cytoplasm where their translation is carried out, lncRNAs are more often located in the nucleus (Kopp and Mendell 2018), where they could play a role in chromatin and genomic structural remodeling, RNA stabilization, and transcriptional regulation (Ransohoff et al., 2018; Rinn and Chang 2012; Takahashi et al., 2014a; Taniue et al., 2016b). However, several lncRNAs are also found specifically in the cytoplasm (Kino et al., 2010; Yoon et al., 2012), where they regulate protein stability by preventing post-translational modifications associated with protein degradation (Taniue et al., 2016a; Yoon et al., 2013). Moreover, lncRNA can also be involved in mRNA and protein localization (Yanagida et al., 2013). Furthermore, lncRNA works as a decoy that precludes the access of regulatory proteins to DNA to repress gene expression, and functions as a guide to recruit chromatin-modifying enzymes to target genes in cis and trans (Wang and Chang 2011). Besides, lncRNA can act as a scaffold to bring two or more proteins into discrete complexes (Wang and Chang 2011; Rinn and Chang 2012). Some of the lncRNAs work as competitive endogenous RNAs (ceRNAs), or miRNA sponges, to bind to the complementary site of targeted miRNAs and regulate their expression and activity to modulate their downstream pathway (Poliseno et al., 2010; Thomson and Dinger 2016).

Although the underlying mechanism of lncRNAs in cancer development is not elucidated, recent studies have demonstrated the functional significance of lncRNAs in cell proliferation, invasion, metastasis, and resistance to adverse stresses, such as hypoxia or chemotherapy (Duguang et al., 2017; Pandya et al., 2020; Takahashi et al., 2014a). LncRNAs play crucial oncogenic and tumor-suppressive roles in several cancers. For example, the lncRNA *H19*, induced during the development of the liver in mice (Pachnis et al., 1988), was highly expressed in hepatocellular carcinoma (HCC) tissues and could induce drug resistance in HCC (Iizuka et al., 2002; Tsang and Kwok 2007). Several recent reports also provide essential insights into the molecular functions, mechanisms, and involvement of lncRNA in PDAC (see *The Functional Roles of Selected lncRNA in EMT of PDAC* section).

## EMT and PDAC

EMT is intimately associated with the development of tissues or organs during embryogenesis. Additionally, this phenomenon is closely correlated with tumor development (O’Brien et al., 2020) and is a trigger for invasion, migration, and acquisition of stem cell-like phenotype in cells of diverse cancers, including PDAC (De Craene and Berx 2013; Zhou et al., 2017). EMT promotes the gain of epithelial stem cell properties, association with stem-like cell markers, and generation of cancer stem cells (Mani et al., 2008). Pancreatic cancer stem cells (PCSCs) promote tumor growth and progression through several mechanisms, including tumor-initiation by inducing stem cell markers CD44, CD24, and CD133, and evasion of conventional therapies (Zhou et al., 2017). PCSCs are correlated with various processes, such as elevated expression of ATP-binding cassette transporter and anti-apoptotic proteins, induction of aldehyde dehydrogenase activity, and enhancement of DNA damage checkpoint repair. These regulatory mechanisms, which can protect cancer cells and enhance the cell survival signaling pathways, cause resistance to chemotherapy and radiotherapy (Baumann et al., 2008; Holohan et al., 2013).

EMT is established by EMT-inducible transcription factors, such as Snail, Slug, ZEB1, ZEB2, and Twist. These transcription factors repress the epithelial marker E-cadherin and upregulate the mesenchymal marker N-cadherin to promote EMT (Nieto and Cano 2012), and are regulated by several diverse upstream regulators, including signaling molecules and ncRNAs by various mechanisms. While many miRNAs modulate the EMT pathway, only a handful of lncRNAs affect EMT in PDAC. Among the well-known oncogenic lncRNAs, *H19*, HOX antisense intergenic RNA (*HOTAIR*), *MALAT1*, long intergenic non-protein coding RNA, regulator of reprogramming (*linc-ROR*), and HOXA transcript at the distal tip (*HOTTIP*) can enhance EMT (Duguang et al., 2017; Fu et al., 2017a; Fu et al., 2017b; Kim et al., 2013; Ma et al., 2016). Moreover, previously unrecognized lncRNAs, *LOC389641* or *ENST00000480739*, are also shown to promote EMT (Sun et al., 2014; Zheng et al., 2016). Collectively, these reports elucidate that lncRNAs may have essential functions in the EMT pathway and gain of stem cell-like properties in PDAC. The individual roles of characterized lncRNAs are reviewed in the following section.

## The Functional Roles of Selected lncRNA in EMT of PDAC

**FIGURE 1:**
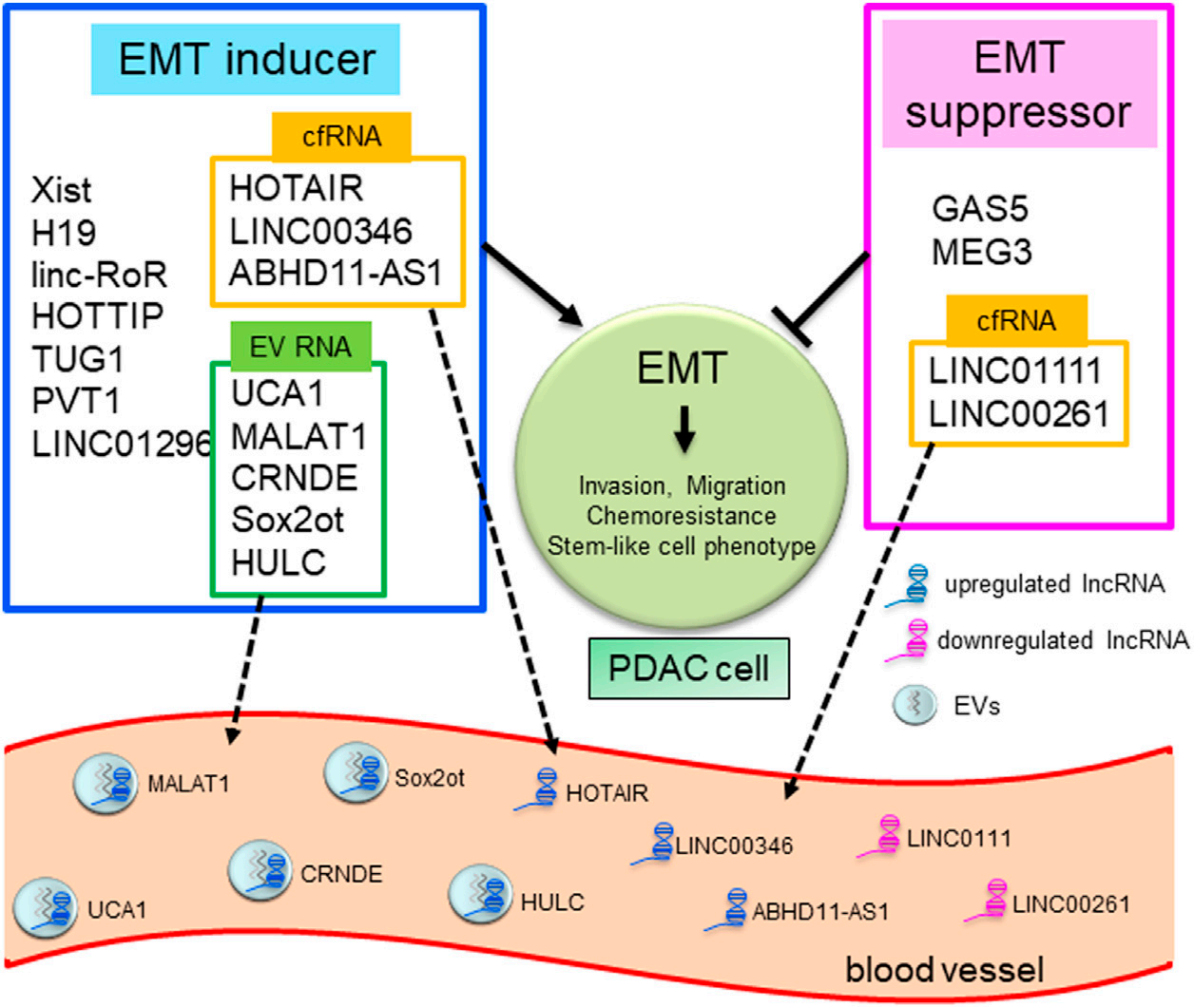
LncRNAs in EMT and liquid biopsy in human PDAC. Schematic overview showing lncRNAs mediating the EMT pathway and as tools for liquid biopsy in PDAC. LncRNAs as EMT inducers or suppressors are summarized in blue or red frames. LncRNAs in yellow or green frames are reported to exist in blood as EV-encapsulated RNAs or cell-free RNAs (cfRNAs) and can be used for liquid biopsy.

LncRNAs have diverse functions to mediate tumor cell progression, including the EMT process. Over recent decades, several lncRNAs have been reported to contribute to PDAC development, primarily through epigenetic regulation of EMT. Here, the crucial lncRNAs that can promote or suppress tumor invasion and metastasis by EMT regulation in PDAC are summarized (Duguang et al., 2017; Pandya et al., 2020) (Table 1; Figure 1). Note, the following lncRNAs are considered oncogenic in PDAC: X inactive specific transcript (*Xist*), *H19*, *UCA1*, *linc-RoR*, SOX2 overlapping transcript (*Sox2ot*), *MALAT-1*, *HOTAIR*, *HOTTIP*, colorectal neoplasia differentially expressed (*CRNDE*), taurine upregulated gene 1 (*TUG1*), plasmacytoma variant translocation 1 (*PVT1*), *HULC*, *LINC01296*, and *LINC00346*. Meanwhile, the following are considered to be oncosuppressive lncRNAs: growth arrest-specific 5 (*GAS5*), maternally expressed gene 3 (*MEG3*), *LINC01111*, and *LINC00261*. The molecular mechanisms, as well as the targeted, or related, genes of these lncRNAs are summarized in Table 1. Reviews are generally cited in other reviews only when referring to specific perspectives offered by them.

**TABLE 1:** LncRNAs in PDAC development through EMT regulation.

lncRNA	Locus	Molecular mechanisms	EMT-related roles in PDAC	References
Xist (X inactive specific transcript)	Xq13.2	down: miR-141-3p	act as a sponge to miR-141-3p enhance invasion and migration	Sun and Zhzng. (2019)
H19	11q15.5	up: E2F-1, HMGA2	promote cell proliferation and cell cycle by E2F-1 upregulation enhance cell invasion and migration by induction of HMGA2	Ma et al. (2014), Ma et al. (2016)
UCA1 (urothelial carcinoma-associated 1)	19q13.12	up: AMOTL2, p-ERK1/2	act as a sponge to miR-96-5p promote angiogenesis *via* upregulation of AMOTL2 and phospho-ERK1/2	Guo et al. (2020)
		down: miR-96–5p		
linc-RoR (long intergenic non-protein coding RNA, regulator of reprogramming)	20q11.23	up: ZEB1	upregulate EMT regulator, ZEB1 act as a sponge to let-7 family promote cell invasion and metastasis	Zhan et al. (2016), Fu et al. (2017a)
		down: let-7		
Sox2ot (SOX2 overlapping transcript)	3q26.33	up: Sox2	act as a sponge to miR-200 family, gain stem cell like phenotype promote invasion and metastasis	Li et al. (2018)
		down: miR-200 family		
MALAT1 (metastasis associated lung adenocarcinoma transcript 1)	9q21.3	up: CD133, VEGF, Sox2	gain stem cell like phenotype, promote angiogenesis and chemoresistance	Jiao et al. (2015)
HOTAIR (HOX antisense intergenic RNA)	12q13.13	down: GDF15	coordinately act with PRC2, promote cell viability and cell cycle	Kim et al. (2013)
HOTTIP (HOXA transcript at the distal tip)	7p15.2	up: Wnt/β-catenin pathway, HOXA9	highly expressed in PCSCs, promote HOXA9 expression, enhance PCSCs properties through Wnt/β-catenin pathway	Fu et al. (2017a)
CRNDE (colorectal neoplasia differentially expressed)	19p11	up: IRS1	act as a sponge to miR-384, promote cell growth and metastasis *via* upregulation of IRS1	Wang et al. (2017)
		down: miR-384		
TUG1 (taurine upregulated gene 1)	22q12.2	up: EZH2	act as a sponge to miR-382 and upregulate EZH2, promote EMT phenotype, cell migration and proliferation	Zhao et al. (2017)
		down: miR-382		
PVT1 (plasmacytoma variant translocation 1)	8q24.21	up: ULK1	act as a sponge to miR-20a and upregulate ULK1, enhance cytoprotective autophagy and cell growth	Huang et al. (2018)
		down: miR-20a		
HULC (highly upregulated in liver cancer)	6p24.3	up: Snail	act as a sponge to miR-133b and miR-622, promote invasion and migration	Takahashi et al. (2020a), Takahashi et al. (2020b)
		down: miR-133b, miR-622		
LINC (long intergenic long non-coding RNA) 01296	14q11.2	up: Bcl-2	promote cell growth and EMT, and inhibit apoptosis by induction of Bcl-2	Yuan et al. (2019)
		down: Bax		
LINC (long intergenic long non-coding RNA) 00346	13q34	up: BRD4	act as a sponge to miR-188-3p and upregulate BRD4 promote cell proliferation and chemoresistance	Shi et al. (2019)
		down: miR-188-3p		
LOC389641	22q.12.1	up: Vimentin, Snail	promote cell growth and EMT by induction of Snail and Vimentin, reduction of E-cadherin	Zheng et al. (2016)
		down: E-cadherin		
AFAP1-AS1	4p16.1	up: Vimentin, N-cadherin, Snail	promote cell invasion and migration *via* induction of EMT, expression in PDAC tissue predict early tumor recurrence	Ye et al. (2015)
		down: E-cadherin		
LINC (long intergenic long non-coding RNA) 00675	357 bp	up: Vimentin, N-cadherin	promote cell invasion *via* induction of EMT	Li et al. (2015)
		down: E-cadherin	expression in PDAC tissue predict early tumor recurrence	
GAS5 (growth arrest-specific 5)	1q25.1	up: SOCS3	act as a sponge to miR-221, inhibit EMT and stem cell-like properties *via* induction of SOCS3	Liu et al. (2018)
		down: miR-221		
MEG3 (Maternally expressed gene 3)	14q32.2	down: Snail	attenuate EMT and stem cell properties, suppress cell invasion, migration and chemoresistance	Ma et al. (2018)

Some of the first discovered lncRNAs described were *Xist and H19*. *Xist* and *H19* can modulate gene expression *via* targeted effects on chromatin remodeling or X chromosome inactivation and imprinting. Loss of imprinting at the *H19* locus increases *H19* expression in HCC (Matouk et al., 2007; Pachnis et al., 1988). In PDAC tissues, *Xist* is upregulated, and directly targets and attenuates the activity of miR-141-3p, which was downregulated, to enhance tumor cell invasion and migration (Sun and Zhang 2019). *H19* also participates in the tumorigenesis of several cancers. *H19* upregulates E2F-1 expression and promotes pancreatic cancer proliferation *via* cell cycle regulation (Ma et al., 2016). Additionally, *H19* enhances cell invasion and migration involved in EMT induction through expression of HMGA2 and let-7 (Ma et al., 2014). Together, the lncRNAs involved in chromatin remodeling, *Xist* and *H19*, serve as oncogenes in PDAC development.

The oncogenic lncRNA *UCA1* is highly expressed in PDAC tissues and cell-derived EVs, especially under hypoxic conditions. *UCA1* could be transported by EVs, and promotes angiogenesis and tumor growth in PDAC cells under hypoxia. *UCA1* acts as a ceRNA that binds to targeted miRNAs *via* the complementary site, thus, regulating miRNA expression and activity, and modulating the downstream pathways of miR-96-5p (Guo et al., 2020). *Linc-RoR* works as an oncogene in several cancers (Pan et al., 2016), enhances the cellular tolerance to hypoxic stress by acting as a miRNA sponge to miR-145, and promotes chemoresistance to anti-cancer drugs by inducing stem cell-like properties, as shown in HCC (Takahashi et al., 2014b; Takahashi et al., 2014c). It is highly upregulated in PDAC tissues compared to adjacent normal tissues and promotes cell migration and metastasis by regulating a crucial EMT regulator, ZEB1 (Zhan et al., 2016). Additionally, *linc-RoR* acts as a ceRNA to the let-7 miRNA family and induces the properties of cancer stem-like cells (Fu et al., 2017b). Therefore, *linc-RoR* could be an EMT inducer in PDAC cells. LncRNA *Sox2ot* could promote stemness by induction of EMT in PDAC cells. *Sox2ot* acts as a miRNA sponge to the miR-200 family, upregulates Sox2 expression, and promotes EMT and stemness, suggesting that *Sox2ot* can accelerate invasion and metastasis of PDAC (Li et al., 2018). *MALAT-1* is one of the well-known oncogenic lncRNAs that act in diverse tumors, such as lung, hepatocellular or gastric cancers (Fu et al., 2020). (Jiao et al., 2015) reported that *MALAT-1* promotes the EMT process and generates cancer stem cells (CSCs). Acquiring the CSC-like phenotype *via* induction of CD133, VEGF, and SOX2 expression could enhance angiogenesis, tumorigenicity, and resistance to gemcitabine chemotherapy (Jiao et al., 2015). *HOTAIR* is an oncogenic lncRNA reported in colon, liver, breast, and other cancers (Qu et al., 2019). In PDAC, *HOTAIR* is overexpressed in cell lines and tumor tissues. Moreover, *HOTAIR* regulates cell viability and cell cycle by suppressing the promoter activity of the growth-inhibitory and proapoptotic gene GDF15 (Kim et al., 2013). Expression of lncRNA *HOTTIP* is increased in PDAC, which promotes cell growth and EMT (Ghafouri-Fard et al., 2020). Fu et al. (2017a) suggested that *HOTTIP* is highly expressed in PCSCs, promotes HOXA9 expression, and enhances PCSC properties through the induction of the Wnt/β-catenin pathway. This mechanism could facilitate cancer cell progression in PDAC (Fu et al., 2017a).

Moreover, the lncRNA *CRNDE* is also reported to be involved in EMT of PDAC. *CRNDE* expression is upregulated in PDAC cell lines and tissues. It acts as a ceRNA, and directly targets and attenuates miR-384 activity. *CRNDE* increases cell growth and metastasis by suppressing miR-384 and upregulating IRS1, which is a crucial mediator of oncogenic insulin-like growth factor (IGF) signaling and can promote tumor development in several cancers (Wang et al., 2017). Similarly, the lncRNA *TUG1* is aberrantly expressed in many cancers (Kondo et al., 2017). *TUG1* is significantly upregulated in PDAC tissues, competitively working as a sponge to miR-382, and upregulating the expression of enhancer of zeste homolog 2 (EZH2), which is targeted by miR-382. Through the induction of EZH2 expression, *TUG1* could promote EMT, PDAC cell migration, and proliferation (Zhao et al., 2017). *PVT1* is an oncogenic lncRNA that is highly expressed in PDAC tissues and is correlated to a worse prognosis. *PVT1* acts as a ceRNA to miR-20a, and upregulates the expression of Unc-51 like kinase 1 (ULK1) protein by suppressing miR-20a activity. Induction of ULK1 expression by *PVT1* could enhance cytoprotective autophagy and tumor cell growth *in vitro* and *in vivo* (Huang et al., 2018). As an oncogenic lncRNA in PDAC, we previously reported that lncRNA *HULC* was exceptionally expressed in PDAC cell lines and could enhance cell invasion and migration through induction of EMT. *HULC* acts as a miRNA sponge to EMT-suppressive miRNAs miR-133b or miR-622. Downregulation of miR-133b and miR-622 by *HULC* leads to the upregulation of EMT-inducing transcription factor Snail, and cell invasion and migration (Takahashi et al., 2020a; Takahashi et al., 2020b).

Furthermore, previously unknown lncRNAs are shown to be related to the EMT process. *LINC01296* is reported to be highly expressed in some cancers (Xu et al., 2019). Specifically, *LINC01296* is increased in resected PDAC tissues, and its expression is associated with advanced stage, presence of lymph node metastasis, and worse patient prognosis. Knockdown of *LINC01296* inhibits cell growth, enhances apoptosis by modulating Bcl-2 and Bax expression, and suppresses EMT, cell invasion, and migration (Yuan et al., 2019). *LINC00346* is also reported to promote cell proliferation, cell cycle, tumorigenesis, and gemcitabine resistance. *LINC00346* serves as a miRNA sponge for miR-188-3p, inhibiting its activity and upregulates the expression of its target, bromodomain-containing protein 4 (BRD4), in PDAC cells. An increase in BRD4 expression leads to PDAC cell proliferation or chemoresistance (Shi et al., 2019).

In contrast, the lncRNA *GAS5* works as a tumor suppressor in multiple cancers (Shi et al., 2013). *GAS5* acts as a ceRNA for miR-221, which is known to promote carcinogenesis. Downregulation of miR-221 by *GAS5* results in the induction of SOCS3 expression, which inhibits EMT and stemness, thereby attenuating PDAC cell proliferation, migration, and chemoresistance (Liu et al., 2018). *MEG3* is another tumor-suppressive lncRNA (Shi et al., 2013), with a decreased expression in PDAC cell lines and tissues that is associated with an advanced TNM stage and poor prognosis. *MEG3* attenuates EMT and stemness, thereby suppressing cell invasion, migration, and gemcitabine chemoresistance (Ma et al., 2018). Meanwhile, *MEG3* is also reportedly an oncogenic lncRNA. According to the single cell RNA-sequencing (scRNA-seq) transcript data, *MEG3* is highly expressed in metastatic PDAC tumors (Pan et al., 2021). The lncRNA *LINC01111* is downregulated in PDAC tissues and patient-derived plasma, and positively correlates with better prognosis of patients. *LINC01111* inhibits cell cycle, cell invasion and migration *in vitro*, and metastasis *in vivo* by acting as a ceRNA to miR-3924, directly targeting and upregulating dual-specificity phosphatase 1 (DUSP1), and the subsequent suppression of the SAPK/JNK signaling pathway by DUSP1 (Pan et al., 2019). Furthermore, the lncRNA *LINC00261* is also downregulated in PDAC tissues. It is downregulated by TGF-β signaling, and upregulated by the EMT-suppressive transcription factor forkhead box protein A2 (FOXA2) by binding to the *LINC00261* promoter. Altogether, *LINC00261* could be a tumor-suppressive lncRNA as it inhibits EMT (Dorn et al., 2020). These mechanisms can contribute to the suppression of tumor development (Pan et al., 2019). To summarize, *GAS5*, *MEG3*, *LINC0111*, and *LINC00261* are tumor-suppressors in PDAC that act *via* inhibition of EMT.

Circular RNAs (circRNAs) and pseudogenes are categorized as subclasses of lncRNAs. CircRNAs are closed RNA transcripts that are generated by unique back-splicing of precursor mRNAs. However, the mechanisms underlying circRNA biogenesis, as well as the regulatory factors involved in their circularization, are not well understood. CircRNAs reportedly have several biological functions, including interacting with RNA-binding protein (RBP), function as an miRNA sponge, and regulating alternative splicing (Tang et al., 2021). For instance, *circEYA3* is derived from the *EYA3* gene and is highly expressed in PDAC tissues. In fact, elevated *circEYA3* levels correlate with poor prognosis of PDAC patients. Mechanistically, *circEYA3* facilitates PDAC cell invasion and migration *via* induction of EMT by its function as an endogenous miR-1294 sponge (Rong et al., 2021). *CircNEIL3* is reportedly increased in PDAC tissue where it accelerates tumor cell migration and invasion through a sponge effect on miR-432-5p (Shen et al., 2021). These circRNAs regulate EMT and contribute to the promotion of tumor development in PDAC.

Pseudogenes have also been recognized as sophisticated modulators of gene expression. Recent studies demonstrated that a large number of pseudogenes, as well as their variable interactions with other biological molecules, may affect several pathways associated with cancer development (Hu et al., 2018). For instance, pseudogene pituitary tumor-transforming 3 (*PTTG3P*) is upregulated in PDAC tissue and is associated with larger tumor size and reduced overall survival. *PTTG3P* functions as a sponge for miR-132/212-3p, causing an increase in FoxM1 expression as well as PDAC cell invasion and migration (Liu et al., 2020). Moreover, pseudogene *WTAPP1* is also elevated in PDAC tissue and is associated with poor prognosis. Specifically, *WTAPP1* may PDAC cell proliferation and invasion *via* Wnt signaling activation (Deng et al., 2021). Hence, various pseudogenes appear to function as oncogenic genes in PDAC.

While few studies have provided evidence that circulating lncRNA in EVs can regulate the characteristics of recipient cells in distant tissues or organs in PDAC, potential roles for EV-encapsulated miRNAs in inducing systemic instigation in several cancers. For example, adipose tissue-derived EV miRNAs, such as miR-99b or miR-222, have been proposed to contribute to the regulation of metabolic homeostasis. These miRNAs can function in a paracrine, autocrine or endocrine manner (Mori et al., 2019). Moreover, miR 25-3p is reportedly transferred by colorectal cancer cell-derived exosomes to distant organs, such as the liver or lung, and induces pre-metastatic niche formation through the promotion of vascular permeability and angiogenesis in a murine model (Zeng et al., 2018). Furthermore, exosome-encapsulated miR-1247-3p, derived from HCC cells, can activate cancer-associated fibroblasts (CAFs), thereby promoting cancer progression. Indeed, EV miR-1274-3p abundance in serum is significantly associated with lung metastasis in HCC patients (Fang et al., 2018). Collectively, these reports imply that EVs, such as exosomes, mediate the transfer of miRNAs, which can modulate the formation of distant metastasis in many cancer types. Thus, investigation of EV-encapsulated lncRNAs that can act *via* endocrine instigation, are warranted in the future.

Finally, we review the lncRNAs that are correlated with PDAC driver gene mutations. *KRAS* and *TP53* are recognized as driver genes for PDAC, and their mutation is one of the most crucial initiation steps during carcinogenesis (Bailey et al., 2016). A few lncRNAs are reported to be related to the mutations in these driver genes (Di Agostino 2020; Pandya et al., 2020). *LincRNA1611* and HOX antisense intergenic RNA myeloid 1 (*HOTAIRM1*) were highly expressed in human PDAC tissues compared to adjacent normal tissues. While *lincRNA1611* expression is positively correlated with mutation of *TP53* gene (Wang et al.,. 2015a), *HOTAIRM1* expression was positively associated with that of *KRAS* gene (Luo et al., 2019). These lncRNAs could interact with the mutated driver genes in PDAC.

Gene copy number variation has been recently identified as being related to carcinogenesis, and some lncRNAs were detected to have high amplification with one or more copy number alterations (Waddell et al., 2015). Although there are some new topics that are not elucidated in this review, such as the roles of lncRNAs located on high amplified chromosome region in EMT, previously unrecognized gene alterations in lncRNAs that are correlated with PDAC initiation and development must be investigated in future studies.

## Diagnostic and Therapeutic Potential of EMT-Related lncRNAs

The most-used standard biomarker for PDAC diagnosis is carbohydrate antigen 19-9 (CA19-9). However, the CA19-9 positive ratio is about 55% for stage I cancers, suggesting that CA19-9 might not be a satisfactory biomarker for the early detection of PDAC (Liu et al., 2012). Therefore, it is necessary to identify a feasible and reliable marker supporting diagnosis at the initial stage of PDAC. LncRNAs have been reported as valuable liquid biopsy tools for PDAC diagnosis using serum, plasma, and other body fluids (Wang et al., 2015b). Recently, circulating tumor cells (CTCs), cell-free DNA (cfDNA), cell-free RNA (cfRNA), and EVs have all been investigated as potential tools for liquid biopsy (Crowley et al., 2013; Yoshioka et al., 2018). Some lncRNAs can be detected in cfRNA or EVs in body fluids and may serve as biomarkers for diagnosing PDAC. The EMT-related lncRNAs in serum or plasma that can serve as potential biomarkers for human PDAC are summarized in Table 2. *HOTAIR*, *LINC00346*, *ABHD11-AS1*, *LINC01111*, and *LINC00261* in serum or plasma, are proposed to be valuable biomarkers for PDAC, while *UCA1*, *MALAT1*, *CRNDE*, *SOX2OT*, and *HULC* are highly expressed in EVs derived from patient blood.

**TABLE 2:** LncRNAs as tools of liquid biopsy for PDAC.

lncRNA	Type of clinical sample	Potential roles for liquid biopsy for PDAC diagnosis	References
HOTAIR	Serum	Increased in PDAC patients compared to healthy individuals	Ma et al. (2019)
		The AUC value for PDAC diagnosis was 0.93	
LINC00346	Serum	Highly expressed in PDAC patients and associated with TNM stage	Zhang et al. (2018)
		The AUC value for PDAC diagnosis was 0.71	
ABHD11-AS1	Plasma	Increased in PDAC patients compared to chronic pancreatitis patients or healthy individuals	Liu et al. (2019)
		The AUC was 0.89 for discriminating PDAC from healthy subjects	
LINC01111	Plasma	Expressed at the low level in PDAC patient	Pan et al. (2019)
		Positively correlated with the good prognosis and overall survival	
LINC00261	Serum	Expressed at the low level in PDAC patient	Zhang et al. (2018)
		Positively correlated with the good prognosis and survival	
UCA1	Serum derived EVs	Increased in PDAC patients compared to healthy individuals	Guo et al. (2020)
		Correlated with poor prognosis of PDAC patients	
MALAT1	Serum derived EVs	Increased in PDAC patients compared to IPMN patients or healthy individuals	Kumar et al. (2020)
CRNDE	Serum derived EVs	Increased in PDAC patients compared to IPMN patients or healthy individuals	Kumar et al. (2020)
Sox2ot	Plasma derived EVs	Highly expressed in PDAC patients and associated with TNM stage	Li et al. (2018)
		Decreased in PDAC patient after operation	
HULC	Serum derived EVs	Increased in PDAC patients compared to IPMN patients or healthy individuals	Takahashi et al. (2020a)
		The AUC was 0.92 for discriminating PDAC from non-PDAC	

Upon analyzing cfRNA in serum or plasma, oncogenic lncRNA *HOTAIR* was found to be highly expressed in serum and tissues from patients with PDAC compared to healthy individuals and adjacent normal tissues. Receiver operating characteristic (ROC) curve analysis with area under the curve (AUC) showed that *HOTAIR* could promote the accuracy of diagnosis for PDAC (Ma et al., 2019). Similarly, *LINC00346* was highly expressed in tissues and serum of patients with PDAC. It was positively correlated with advanced clinical stage and poor survival (Zhang et al., 2018). Additionally, lncRNA *ABHD11-AS1* was found to contribute to the early diagnosis of PDAC. Plasma levels of *ABHD11-AS1* were significantly higher than in patients with chronic pancreatitis than healthy individuals. Moreover, the combination of *ABHD11-AS1* and CA19-9 was found to be more effective for early PDAC diagnosis (Liu et al., 2019). Therefore, these lncRNAs could be potential markers for the early diagnosis of PDAC from patient blood.

In contrast, *LINC01111* was expressed at low levels in the plasma and tissues of patients with PDAC compared to healthy controls or adjacent healthy tissue. The expression level of *LINC01111* was negatively associated with the advanced TNM stage, and positively correlated with favorable prognosis and overall survival in patients with PDAC (Pan et al., 2019). Moreover, *LINC00261* expression, associated with a higher survival rate (Zhang et al., 2018), was decreased in PDAC tissues and patient serum.

There is limited literature regarding lncRNAs encapsulated within EVs being considered as biomarkers for PDAC diagnosis. *UCA1* is highly expressed in PDAC cell-derived EVs and could be transferred from donor to recipient cells by EVs. *UCA1* expression was significantly increased in EVs derived from serum of patients with PDAC compared to healthy donors and was associated with a poor prognosis in these patients (Guo et al., 2020). *MALAT-1* can decrease the chemosensitivity and accelerate tumor angiogenesis by enhancing stem cell-like phenotypes *via* induction of EMT (Jiao et al., 2015), and *CRNDE* promotes cell proliferation, invasion, and migration by sponging miR-384 in PDAC cells (Wang et al., 2017). These two lncRNAs were highly expressed in serum EVs from patients with PDAC compared with controls, suggesting that they could act as crucial liquid biopsy tools for PDAC diagnosis (Kumar et al., 2020). *Sox2ot* promotes PDAC invasion and metastasis by induction of EMT. Plasma EVs containing *Sox2ot* were highly expressed in patients with PDAC and were associated with the advanced TNM stage.

Interestingly, exosomal *Sox2ot* expression was decreased after surgery in the plasma of patients with PDAC (Li et al., 2018). Furthermore, we have recently reported that *HULC* was encapsulated and carried by PDAC cell-derived EVs in body fluids. *HULC* expression in serum EVs was significantly increased in patients with PDAC compared to patients with intraductal papillary mucinous neoplasm (IPMN) or healthy individuals. The ROC curve revealed that EV-encapsulated *HULC* showed good predictive performance, with an AUC of 0.92, when compared to CA19-9 (0.9) and carbohydrate antigen 19-9 (CEA) (0.54) for discriminating against patients with PDAC from those without PDAC (Takahashi et al., 2020a). These reports illustrate the usefulness of lncRNAs in EVs as novel tools for early PDAC diagnosis using liquid biopsy.

Additionally, one study has described the usefulness of lncRNAs as biomarker for IPMN. Specifically, plasma expression of lncRNA *GAS5* and *SRA* has proven useful as markers to distinguish IPMN patients from healthy controls. Moreover, the combined expression of eight lncRNAs (*ADARB2-AS1*, *ANRIL*, *GLIS3-AS1*, *LINC00472*, *MEG3*, *PANDA*, *PVT1*, and *UCA1*) may effectively discriminate between malignant and benign IPMN (Permuth et al., 2017).

Various lncRNAs have also been reported as predictors of metastasis following surgical resection of the primary tumor. For instance, lncRNA *AFAP1-AS1* is overexpressed in human PDAC tissue and can promote PDAC cell invasion and migration *via* induction of EMT. Moreover, elevated expression of *AFAP1-AS1* can predict poor prognosis and tumor recurrence within 6 months and 1 year after surgical resection, respectively (Ye et al., 2015). *LINC00675* is also aberrantly overexpressed in human PDAC tissue and accelerates PDAC cell migration. This upregulation of *LINC00675* in PDAC tissue can predict recurrence within 6 months of surgical resection (Li et al., 2015). Hence, these two lncRNAs may represent effective predictors for early recurrence of PDAC following surgical intervention.

Regarding clinical application for cancer therapy, several studies have investigated lncRNAs as potential therapeutic tools as they could be helpful in advancing therapeutic strategies to target cancer cells. There are some attempts being made to regulate lncRNAs, focusing on their degradation and the functional suppression by use of siRNA, shRNA, or inhibition of transcription (Pandya et al., 2020). EV-encapsulated lncRNAs can also be exploited for clinical applications. An example of EV-mediated gene therapy, the effect of miRNA from human mesenchymal stem cell (hMSC)-derived EVs, was reported in PDAC cells. The transfer of EVs from hMSCs abundant in miR-143-3p, a tumor suppressor miRNA, to PDAC cells attenuated cell invasion and induced apoptosis by suppressing the lncRNA *P11-363N22.3* (Wang et al., 2021). Moreover, transfer of the mutated protein Survivin-T34A by EVs enhanced gemcitabine sensitivity of PDAC cells (Aspe et al., 2014). These reports indicate that EVs would have enormous potential as the transfer tool for nucleic acids, including lncRNA and miRNA (Ariston Gabriel et al., 2020). Even though a majority of these results were based on assays in cell lines, these insights into lncRNAs might provide possible therapeutic applications in future, in addition to diagnostic liquid biopsies that predict prognosis and response to therapy in patients with PDAC (Figure 1).

## Perspectives

The challenge of revealing the molecular mechanisms of EMT in pancreatic cancer that are regulated by lncRNAs could direct the discovery of new therapies, and tools for liquid biopsy to diagnose and predict therapeutic effects. Understanding the regulation of lncRNA expression during the initiation and progression of PDAC would provide a new paradigm on cancer pathogenesis. These biological insights may contribute to a rapid progress in development of diagnostic and therapeutic interventions for PDAC using lncRNA and EV-based strategies. Further studies will be required to validate the utility of the candidate lncRNAs for future clinical applications.
